# Amyloid β‐induced elevation of O‐GlcNAcylated c‐Fos promotes neuronal cell death

**DOI:** 10.1111/acel.12872

**Published:** 2018-12-04

**Authors:** Heesun Choi, Chaeyoung Kim, Hyundong Song, Moon‐Yong Cha, Hyun Jin Cho, Sung Min Son, Haeng Jun Kim, Inhee Mook‐Jung

**Affiliations:** ^1^ Department of Biochemistry and Biomedical Sciences Seoul National University, College of Medicine Seoul Korea

**Keywords:** Alzheimer’s disease, c‐Fos, glucose metabolism, neuronal cell death, O‐linked β‐N‐acetyl glucosamine (O‐GlcNAc), β‐amyloid (Aβ)

## Abstract

Alzheimer's disease (AD) is an age‐related neurodegenerative disease characterized by progressive memory loss resulting from cumulative neuronal cell death. O‐linked β‐N‐acetyl glucosamine (O‐GlcNAc) modification of the proteins reflecting glucose metabolism is altered in the brains of patients with AD. However, the link between altered O‐GlcNAc modification and neuronal cell death in AD is poorly understood. Here, we examined the regulation of O‐GlcNAcylation of c‐Fos and the effects of O‐GlcNAcylated c‐Fos on neuronal cell death during AD pathogenesis. We found that amyloid beta (Aβ)‐induced O‐GlcNAcylation on serine‐56 and 57 of c‐Fos was resulted from decreased interaction between c‐Fos and O‐GlcNAcase and promoted neuronal cell death. O‐GlcNAcylated c‐Fos increased its stability and potentiated the transcriptional activity through higher interaction with c‐Jun, resulting in induction of Bim expression leading to neuronal cell death. Taken together, Aβ‐induced O‐GlcNAcylation of c‐Fos plays an important role in neuronal cell death during the pathogenesis of AD.

## 1 INTRODUCTION

Alzheimer's disease (AD) is an age‐related neurodegenerative disease, characterized by extracellular senile plaques and intracellular neurofibrillary tangles in the brain that causes a progressive cognitive decline and neuronal cell death (Kumar, Singh, & Ekavali 2015,; Querfurth & LaFerla, 2010). Many researches showed that β‐amyloid peptides (Aβ), composing senile plaques, induce cytotoxicity such as cell death (Querfurth & LaFerla, 2010). It has been reported that c‐Fos, one of cell death regulating proteins, is increased in the brains of patients with AD (Marcus et al., 1998). c‐Fos is a transcription factor that binds to the AP‐1 site of DNA by forming a complex with members of the Jun family of proteins (Hess, Angel, & Schorpp‐Kistner, 2004). Although target genes of AP‐1 complex are involved in various processes dependent on specific stimuli (Hess et al., 2004), it is well‐known that AP‐1 complex transcribes apoptotic genes under cytotoxic conditions (Chen et al., 2015; Fernandez et al., 2005; Gillardon et al., 1996; Shaulian & Karin, 2002; Whitfield, Neame, Paquet, Bernard, & Ham, 2001; Zhang et al., 2002). However, the specific mechanism underlying the role of c‐Fos in the pathophysiology of AD is unclear.

Disrupted glucose metabolism is one of pathological features of AD (Schubert, 2005). O‐linked β‐N‐acetyl glucosamine (O‐GlcNAc) modification that reflects glucose metabolism is disrupted in the brains of patients with AD (Alfaro et al., 2012; Forster et al., 2014; Wang et al., 2017; Zhu, Shan, Yuzwa, & Vocadlo, 2014). The O‐GlcNAc modification, regulated by O‐GlcNAc transferase (OGT, an enzyme that adds O‐GlcNAc to proteins from Uridine diphosphate N‐acetylglucosamine (UDP‐GlcNAc)) and O‐GlcNAcase (OGA, and enzyme that removes O‐GlcNAc from proteins), is a dynamic process, affected in various human diseases as well as many biological processes (Bond & Hanover, 2015; Yuzwa & Vocadlo, 2014). A recent studies on proteomic analysis using the brains of AD patients and 3xTg‐AD mice showed altered abundance of O‐GlcNAc peptides in AD (Alfaro et al., 2012; Forster et al., 2014; Wang et al., 2017). Therefore, disrupted O‐GlcNAc modification is considered to be important for understanding the pathophysiology of AD. However, whether dysregulated O‐GlcNAc cycling might be resulted from altered activity or substrate specificity of OGT and OGA in AD remains unclear. In addition, the role of O‐GlcNAc modification in cell death associated with AD has not been studied so far. Although c‐Fos is known to be an O‐GlcNAcylated protein (Tai, Khidekel, Ficarro, Peters, & Hsieh‐Wilson, 2004), the specific dynamics and the effects of c‐Fos O‐GlcNAcylation require further investigation.

Here, we identified the O‐GlcNAcylation sites on c‐Fos and observed an increase in O‐GlcNAcylation on c‐Fos under conditions simulating AD, specifically in 5xFAD mouse which is well‐known AD animal model and Aβ‐treated neurons. In addition, we revealed that Aβ‐induced O‐GlcNAcylation on c‐Fos promotes neuronal cell death by inducing the expression of apoptotic protein Bim, by elevating the transcriptional activity of c‐Fos and increasing its stability. These novel findings contribute to understanding the link between glucose metabolism and neuronal cell death in the pathophysiology of AD.

## 2 RESULTS

### 2.1 Aβ increases c‐Fos O‐GlcNAcylation as well as the protein of c‐Fos

**Figure 1:**
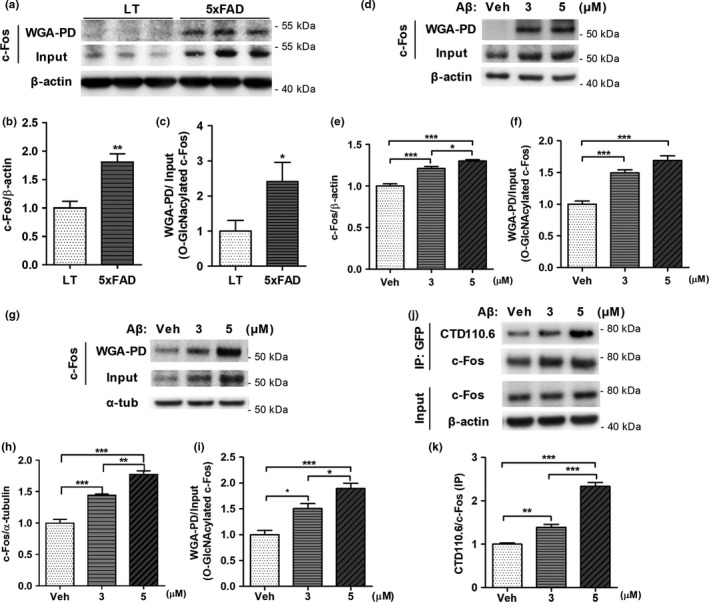
The levels of c‐Fos O‐GlcNAcylation are increased in 5xFAD mice and Aβ treated cells. (a–c) Increased O‐GlcNAcylation of c‐Fos as well as the total levels of c‐Fos in 5xFAD. Representative immunoblot images are shown in (a). Quantitative graph showing the levels of c‐Fos (b) and those of O‐GlcNAcylation of c‐Fos (c) were normalized to the amount of β‐actin and the input level of c‐Fos, respectively. (*n* = 5 per group). Data are shown as mean ± *SEM*. *p < 0.05, **p < 0.01, (Student's *t*‐test). (d–i) Increased O‐GlcNAcylation of c‐Fos as well as the total levels of c‐Fos by Aβ in primary cortical neurons (*n* = 5) (d–f) and SH‐SY5Y cells (*n* = 4) (g–i). Representative immunoblot images (d, g), quantitative graph of the levels of c‐Fos (e, h) and those of O‐GlcNAcylation of c‐Fos (f, i) are shown. Data are presented as mean ± *SEM*. *p < 0.05, **p < 0.01, ***p < 0.001 (one‐way ANOVA, Bonferroni posthoc test). (j, k) Increased level of O‐GlcNAcylation of exogenous c‐Fos by Aβ in SH‐SY5Y cell lines. Representative immunoblot images (j), quantitative graph showing the O‐GlcNAcylation level of c‐Fos detected by O‐GlcNAc specific antibody (CTD110.6) and normalized to the amount of immunoprecipitated EGFP‐c‐Fos (k) (*n* = 4). Data are presented as mean ± *SEM*. *p < 0.05, **p < 0.01, ***p < 0.001 (one‐way ANOVA, Bonferroni posthoc test). α‐tub: α‐tubulin; IP: Immunoprecipitation; LT: wild‐type littermate; Veh: Vehicle; WGA‐PD: wheat‐germ‐agglutinin‐pull down

Several reports show that the expression c‐Fos is increased in the brains of patients with AD, and that c‐Fos can be O‐GlcNAcylated (Marcus et al., 1998; Tai et al., 2004). Since O‐GlcNAc cycling is altered in patients with AD (Forster et al., 2014; Liu et al., 2009; Zhu et al., 2014), we hypothesized that c‐Fos O‐GlcNAcylation might be dysregulated in AD and play a role in its pathogenesis. To determine whether c‐Fos O‐GlcNAcylation is altered under conditions simulating AD, we pulled down O‐GlcNAcylated proteins using wheat‐germ‐agglutinin (WGA)‐conjugated agarose beads (that well‐known to bind to O‐GlcNAc) in the brains of 5xFAD mice, a model of AD, and Aβ‐treated primary cortical neurons and SH‐SY5Y cells. Although the levels of c‐Fos were increased in 5xFAD as reported previously (Marcus et al., 1998), we observed that c‐Fos O‐GlcNAcylation was also increased in 5xFAD compared to wild‐type littermates, which was confirmed when the levels of pulled‐down c‐Fos (O‐GlcNAcylated c‐Fos) were normalized to the input level of c‐Fos (Figure 1a–c). We also found that both c‐Fos and c‐Fos O‐GlcNAcylation levels were increased by Aβ in primary neurons and SH‐SY5Y cells (Figure 1d–i). In addition, when removing O‐GlcNAc using β‐hexosaminidase (β‐HEX), an O‐GlcNAc‐specific exoglycosidase that catalyzes the hydrolysis of β‐D‐N‐acetylglucosamine residues, WGA‐pulled‐down c‐Fos were remarkably decreased in the brains of 5xFAD mice and in the presence of Aβ, although the total levels of c‐Fos levels were not changed (Supporting Information Figure S1). Especially, increased WGA‐pulled‐down c‐Fos by Aβ were reduced even lower than vehicle group by β‐HEX reaction, though, the input level of c‐Fos was not changed (Supporting Information Figure S1b). These data support that c‐Fos O‐GlcNAcylation was increased by Aβ.

Furthermore, to exclude the possibility of c‐Fos induction by Aβ and confirm an increase in c‐Fos O‐GlcNAcylation, we performed immunoprecipitation of overexpressed exogenous c‐Fos using an anti‐GFP antibody (Figure 1j,k). The increase in signals using CTD110.6 antibody, that specifically detects O‐GlcNAc, clearly showed that c‐Fos O‐GlcNAcylation was increased by Aβ.

In addition, we proved that there were no nonspecific bindings during pull down assay and immunoprecipitation assay. To confirm nonspecific binding to beads, cell lysates were incubated with agarose beads, because we use WGA‐coated‐agarose beads when performing WGA‐pull down assay, and there were no signals (2nd lane in Figure 3a and 1st lane in Supporting Information Figure S1b). Competitive assay using exogenously added GlcNAc to cell lysates, to confirm nonspecific binding to WGA, showed that O‐GlcNAcylated c‐Fos binds specifically to WGA (3rd and 4th lane in Figure 3a). In addition,no signal of WGA‐pulled‐down EGFP and no signal of CTD110.6 in immunoprecipitated EGFP (Supporting Information Figure S2) showed that EGFP did not affect the results of confirming c‐Fos O‐GlcNAcylation. From these results, we demonstrated that Aβ can increase the O‐GlcNAcylation of c‐Fos, which reflects dysregulated O‐GlcNAc cycling, as well as increases the level of c‐Fos.

### 2.2 Aβ modulates the interaction between O‐GlcNAcase and c‐Fos

**Figure 2:**
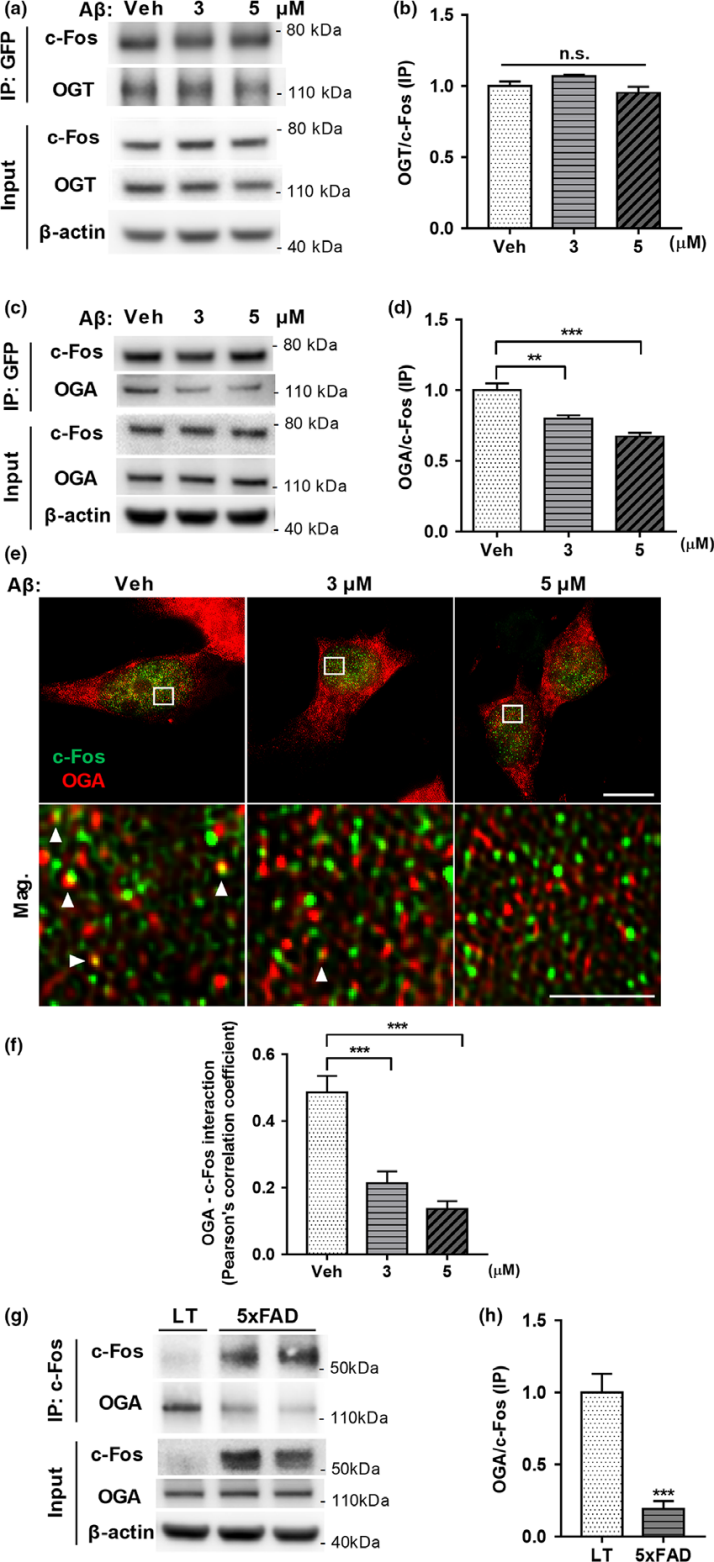
Aβ modulates the interaction between OGA and c‐Fos. (a, b) The interaction between OGT and c‐Fos were not changed by Aβ. Representative immunoblot images (a), quantitative graph showing the levels of co‐immunoprecipitated OGT normalized to the amount of immunoprecipitated EGFP‐c‐Fos (b) (*n* = 4) (c–f) Decreased interaction between OGA and c‐Fos in the presence of Aβ. Representative immunoblot images (c), quantitative graph showing the levels of co‐immunoprecipitated OGA normalized to the amount of immunoprecipitated EGFP‐c‐Fos (d) (*n* = 5). (e, f) The interaction between c‐Fos (green) and OGA (red) were analyzed by SIM. Representative SIM images showing original magnification images in upper panel (100×, scale bar: 5 μm) and magnified images of white boxes in lower panel (scale bar: 1 μm) (e). Quantitatitve graph (f) showing the altered interaction between OGA and c‐Fos in the presence of Aβ by using Pearson's correlation coefficient (*n* = 14, 16 and 12 cells for veh, 3 and 5 μM group, respectively). Arrow heads indicate merged signals. Data are presented as mean ± *SEM*. **p < 0.01, ***p < 0.001 (one‐way ANOVA, Bonferroni posthoc test). (g, h) Decreased interaction between OGA and c‐Fos in 5xFAD mice compared to wild‐type littermate (LT). Representative immunoblot images (c), quantitative graph showing the levels of co‐immunoprecipitated OGA normalized to the amount of immunoprecipitated c‐Fos (d) (*n* = 5, 5). Data are presented as mean ± *SEM*. ***p < 0.001 (student *t*‐test). IP: Immunoprecipitation; Mag.: Magnified images; n.s.: nonsignificant; Veh: Vehicle

Next, we wondered how Aβ dysregulates O‐GlcNAc cycling. Forster et al. (2014) showed that the proteins of approximately 50 – 60 kDa and 25 kDa in size show increased O‐GlcNAcylation, while the proteins of over 70 kDa in size show reduced O‐GlcNAcylation in the brains of patients with AD (Forster et al., 2014). This suggested that O‐GlcNAc cycling in AD was altered. Thus, increased O‐GlcNAcylation on c‐Fos, a 55 kDa protein, may be a result of altered O‐GlcNAc cycling in AD. It has been reported that the interaction between OGT and its substrate (ATP synthase 5A) is altered by Aβ (Cha et al., 2015). Therefore, we wondered whether O‐GlcNAc modifying enzymes (OGT or OGA) are involved in increased c‐Fos O‐GlcNAcylation by Aβ. Immunoprecipitation with GFP antibody, followed by Western blotting with c‐Fos and OGT showed that the interaction between OGT and c‐Fos was not changed by Aβ (Figure 2a,b). However, the interaction between OGA and c‐Fos was decreased by Aβ using immunoprecipitation with GFP and Western blotting with c‐Fos and OGA (Figure 2c,d). To confirm the less interaction between OGA and c‐Fos by Aβ, imaging with structured illumination microscopy (SIM), which is a super‐resolution microscopy, was performed. It showed that less merged signals between c‐Fos and OGA in the presence of Aβ (Figure 2e,f). In addition, we also observed that the interaction between c‐Fos and OGA was decreased in the brains of 5xFAD mice compared to wild‐type littermate (Figure 2g,h). This suggests that the ability of OGA to interact with c‐Fos is altered by Aβ, resulting in increased O‐GlcNAcylated c‐Fos in the cells.

### 2.3 The mapping for O‐GlcNAc sites of c‐Fos

**Figure 3:**
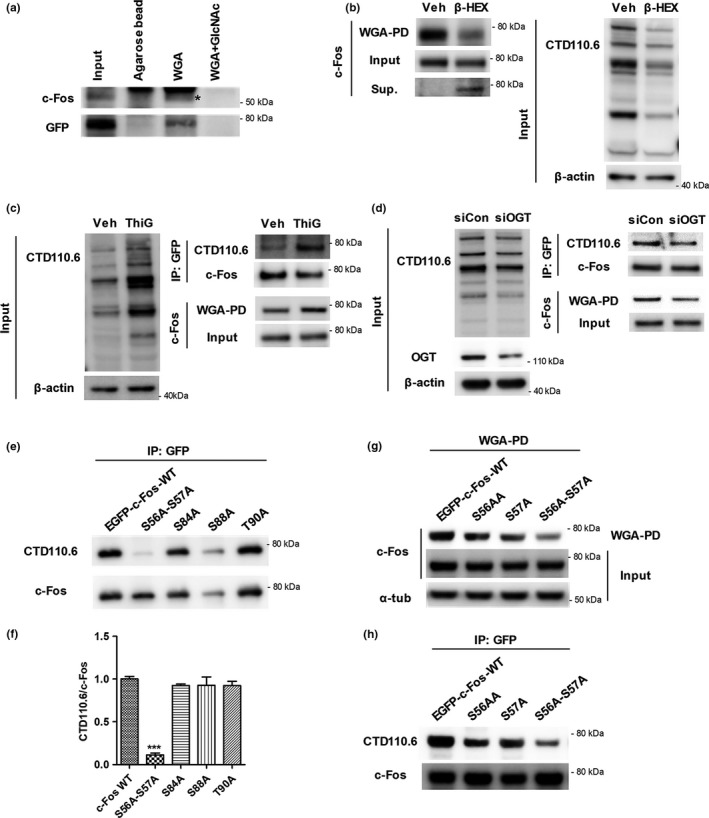
c‐Fos is an O‐GlcNAcylated protein and O‐GlcNAcylated at the sites S56 and S57. (a–d) Endogenous or EGFP‐tagged exogenous c‐Fos from SH‐SY5Y cell lysates was pulled down by using WGA or anti‐GFP antibody and analyzed using Western blotting. (a) Competitive assay showing that both endogenous and exogenous c‐Fos specifically bind to WGA. Both endogenous and exogenous c‐Fos were pulled down using agarose beads or WGA or WGA with GlcNAc. c‐Fos and GFP indicate endogenous and exogenous c‐Fos, respectively. The star indicates a c‐Fos band. (b) β‐hexosaminidase (β‐HEX) assay showing that c‐Fos is O‐GlcNAcylated. Total cell lysates and WGA‐pulled‐down‐O‐GlcNAcylated proteins were incubated with β‐HEX overnight at 37°C. Supernatants of WGA‐pulled‐down samples (Sup.) contain proteins that were de‐O‐GlcNAcylated by β‐HEX. (c, d) c‐Fos O‐GlcNAcylation was regulated by modulating OGA (c) and OGT (d). O‐GlcNAcylated c‐Fos was pulled down using WGA and c‐Fos was immunoprecipitated using an anti‐GFP antibody in Thiamet G (ThiG), an OGA inhibitor, treated cell lysates (c) and siOGT‐transfected cell lysates (d). Immunoblotting with CTD110.6, an O‐GlcNAc specific antibody, demonstrating the efficacy of the treatment. (e–h) Identification of O‐GlcNAc sites on c‐Fos using immunoprecipitation (e, f, h) and WGA‐pull down assay (g) in HEK293T cell lines. (e, f) EGFP‐c‐Fos‐S56A‐S57A show a significantly lower signal compared to other c‐Fos constructs. EGFP‐tagged WT and mutant versions of c‐Fos were immunoprecipitated using an anti‐GFP antibody and probed with CTD110.6 (O‐GlcNAc specific antibody). Representative immunoblot images and a quantitative graph normalized to immunoprecipitated c‐Fos are shown in (e) and (f), respectively (*n* = 3). Data are presented as mean ± *SEM*. ***p < 0.001 (one‐way ANOVA, Bonferroni posthoc test). (g, h) The O‐GlcNAc sites on c‐Fos are S56 and S57. Immunoblot images of a WGA‐pull down assay (g) and immunoprecipitation using an anti‐GFP antibody (h). α‐tub: α‐tubulin; β‐HEX: β‐hexosaminidase; IP: Immunoprecipitation; siCon: control siRNA; ThiG: Thiamet G; Veh: Vehicle; WGA‐PD: wheat‐germ‐agglutinin‐pull down

Tai et al. (2004) reported new methods to find O‐GlcNAc modified proteins. One of examples for low abundance molecules was c‐Fos protein. They did not examine the site mapping of O‐GlcNAc on c‐Fos as well as the function of O‐GlcNAc on c‐Fos at all. In this study, we first confirmed O‐GlcNAcylation of c‐Fos using various experimental designs consistent with commonly used methods. Both endogenous and exogenous c‐Fos were pulled down using WGA. However, when incubated with exogenously added GlcNAc to compete with endogenous GlcNAc within O‐GlcNAcylated proteins, the signal by O‐GlcNAcylated c‐Fos disappeared (Figure 3a). Also, when removing O‐GlcNAc using β‐HEX, the amount of WGA‐pulled‐down c‐Fos was reduced and, consistently, that of de‐O‐GlcNAcylated c‐Fos within the supernatant was increased (Figure 3b). OGA inhibition (more O‐GlcNAc on the protein) or OGT knockdown (less O‐GlcNAc on the protein) also demonstrated that c‐Fos is an O‐GlcNAcylated protein (Figure 3c,d). When OGA was inhibited by Thiamet G, an increase in CTD110.6 signal and the amount of WGA‐pulled‐down c‐Fos was observed. In contrast, a decrease in CTD110.6 signal and the amount of WGA‐pulled‐down c‐Fos was observed when OGT was knocked down with antisense RNA against OGT. These results clearly showed that c‐Fos is an O‐GlcNAcylated protein. We also confirmed that c‐Fos was O‐GlcNAcylated in other cell types such as HEK293T cells (Supporting Information Figure S2).

Next, we identified the sites of O‐GlcNAc modification on c‐Fos using a WGA‐pull down assay and immunoprecipitation. Point mutations were introduced at potential O‐GlcNAc sites to prevent O‐GlcNAcylation by substituting serine (S) or threonine (T) with alanine (A), and each mutant was pulled down using WGA (Supporting Information Figure S3). We established using EGFP‐c‐Fos‐S56A‐S57A, S84A, S88A, and T90A mutants that the S56, S57, S84, S88, and T90 were high potential sites for O‐GlcNAc modifications by performing three repeat experiments. We then used immunoprecipitation with anti‐GFP antibody, followed by Western blotting with CTD110.6 antibody to confirm whether these sites were O‐GlcNAcylated (Figure 3e,f). It showed that EGFP‐c‐Fos‐S56A‐S57A showed a dramatically decreased CTD110.6 signal, but the signal for other mutants did not change. Thus, we predicted that S56 or S57 were the potential sites of O‐GlcNAc, and designed EGFP‐c‐Fos‐S56A and EGFP‐c‐Fos‐S57A mutants to establish the exact site of O‐GlcNAc (Figure 3g,h). In both the WGA‐pull down assay (Figure 3g) and the immunoprecipitation assays using EGFP‐c‐Fos (Figure 3h), O‐GlcNAcylated c‐Fos‐S56A and c‐Fos‐S57A showed lower signals compared to c‐Fos‐WT. Moreover, a decrease in the signals for c‐Fos‐S56A‐S57A was larger compared to those for c‐Fos‐S56A and c‐Fos‐S57A. These data indicated that both S56 and S57 of c‐Fos were O‐GlcNAcylated. Many researchers identified O‐GlcNAc sites using mass spectrometry (LC‐MS/MS) with electron transfer dissociation (ETD). Thus, we have approached the mass spectrometric analysis to confirm c‐Fos O‐GlcNAc sites. However, it turned out that we have not been successful to confirm the O‐GlcNAcylation on S56 and S57 by mass spectrometry with ETD. Probably, because of sequence characteristics of c‐Fos protein, there might be not enough ionization on the peptide around S56 and S57 residues. From these data, we carefully suggested that both S56 and S57 of c‐Fos are the first identified O‐GlcNAc sites on c‐Fos.

### 2.4 O‐GlcNAcylation of c‐Fos promotes cell death in the presence of Aβ

**Figure 4:**
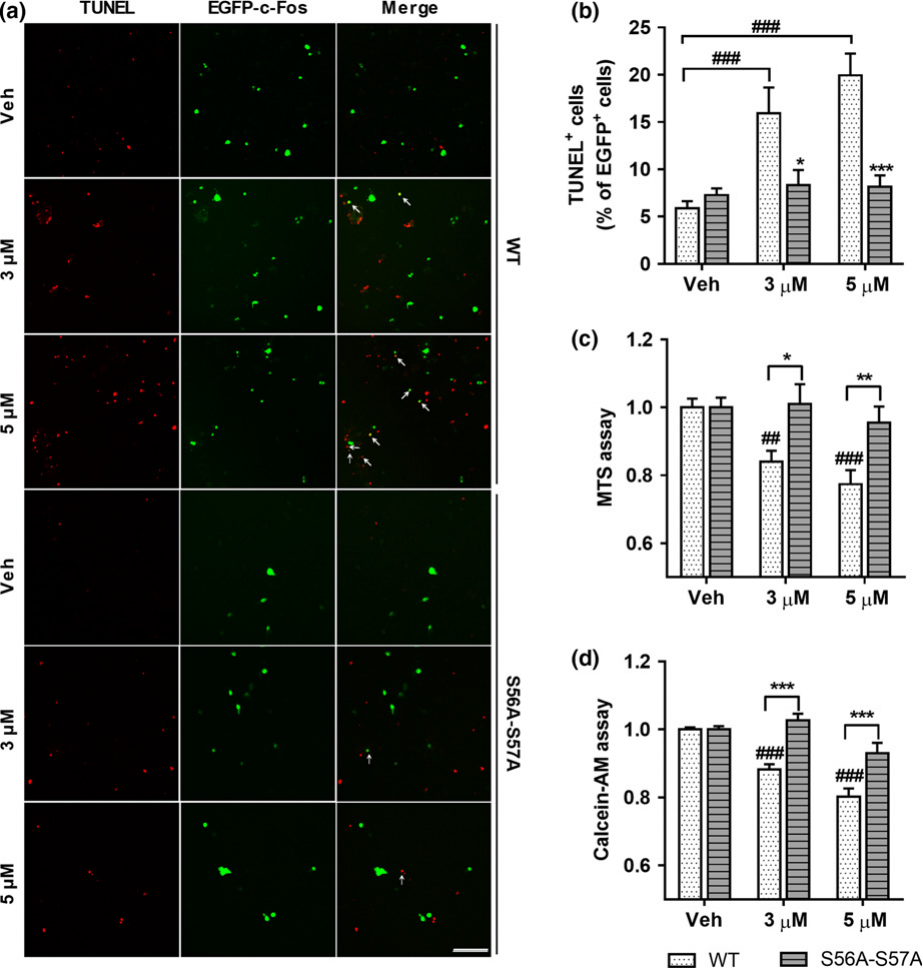
O‐GlcNAcylation of c‐Fos on S56 and S57 promotes neuronal cell death in the presence of Aβ. (a, b) EGFP‐c‐Fos‐WT or EGFP‐c‐Fos‐S56A‐S57A transfected SH‐SY5Y cell lines were treated with Aβ (at doses indicated) for 24 hr. Representative images of TUNEL assay (scale bar: 250 μm) (a) and quantitative graph (b) show that EGFP‐c‐Fos‐S56A‐S57A prevented Aβ‐induced cell death (*n* = 6). Arrows indicate TUNEL^+^ EGFP^+^ cells. (c, d) Tag free‐c‐Fos‐WT or tag free‐c‐Fos‐S56A‐S57A transfected SH‐SY5Y cell lines were treated with Aβ (at doses indicated) for 24 hr. Quantitative graphs of MTS assay (c) and Calcein‐AM assay (d) show that tag free‐c‐Fos‐S56A‐S57A transfected groups were resistant to cell death compared to tag free‐c‐Fos‐WT transfected groups in the presence of Aβ ((c): *n* = 8, (d): *n* = 8). Data are shown as mean ± *SEM*. ^##^ *p* < 0.01, ^###^ *p* < 0.001 among c‐Fos‐WT or c‐Fos‐S56A‐S57A transfected groups (one‐way ANOVA, Bonferroni posthoc test), *p < 0.05, **p < 0.01, ***p < 0.001 between c‐Fos‐WT and c‐Fos‐S56A‐S57A groups (two‐way ANOVA, Bonferroni posthoc test). S56A‐S57A: c‐Fos‐S56A‐S57A; Veh: Vehicle; WT: c‐Fos‐WT

Several studies show that c‐Fos is involved in cell death under cytotoxic conditions because it regulates the transcription of apoptotic genes (Chen et al., 2015; Fernandez et al., 2005; Gillardon et al., 1996; Whitfield et al., 2001). Aβ is a well‐known cytotoxic protein that induces neurite atrophy, synaptic dysfunction, and neuronal cell death (Kumar et al., 2015; Querfurth & LaFerla, 2010). Thus, we hypothesized that c‐Fos may be involved in cell death in response to Aβ and that O‐GlcNAcylation on c‐Fos may regulate cell viability. To determine this, we carried out TUNEL assay for measuring cell death, and MTS and Calcein‐AM assays for measuring cell viability in c‐Fos‐WT or c‐Fos‐S56A‐S57A transfected SH‐SY5Y (Figure 4) and HEK293T cells (Supporting Information Figures S4a,b). When cells were treated acutely with a high dose of Aβ that induces cell death cell death was occurred in EGFP‐c‐Fos‐WT transfected cells. However, O‐GlcNAc incompetent EGFP‐c‐Fos‐S56A‐S57A mutant prevented Aβ‐induced cell death (Figure 4a,b). Consistently, cell viability was decreased in c‐Fos‐WT transfected cells, while in cells transfected with O‐GlcNAc incompetent c‐Fos‐S56A‐S57A mutant, cell viability was significantly restored compared to c‐Fos‐WT transfected cells (Figure 4c,d), suggesting that O‐GlcNAcylated c‐Fos is required for Aβ‐induced neuronal cell death. To confirm that Aβ‐induced cell death is indeed mediated by c‐Fos O‐GlcNAcylation, we performed several further experiments. First, to verifying overexpression effects and what happens endogenously, we compared mock and wild‐type c‐Fos transfected conditions (Supporting Information Figures S4c,d). The results showed that there were no significant differences between mock and wild‐type c‐Fos transfected groups in cell viability assays. Second, to exclude interference of endogenous c‐Fos in Figure 4c,d, we performed cell viability assays using c‐Fos knock‐out stable cell line, showing similar results of regular cell line (Supporting Information Figures S4e–g). This indicated that the effects of endogenous c‐Fos are slight, thus, endogenous c‐Fos may hardly ever interfere the results of Figure 4c,d. Thus, these data demonstrated that O‐GlcNAcylation of c‐Fos on S56 and S57 plays an important role in Aβ‐induced cell death.

### 2.5 O‐GlcNAcylation of c‐Fos increases its stability

**Figure 5:**
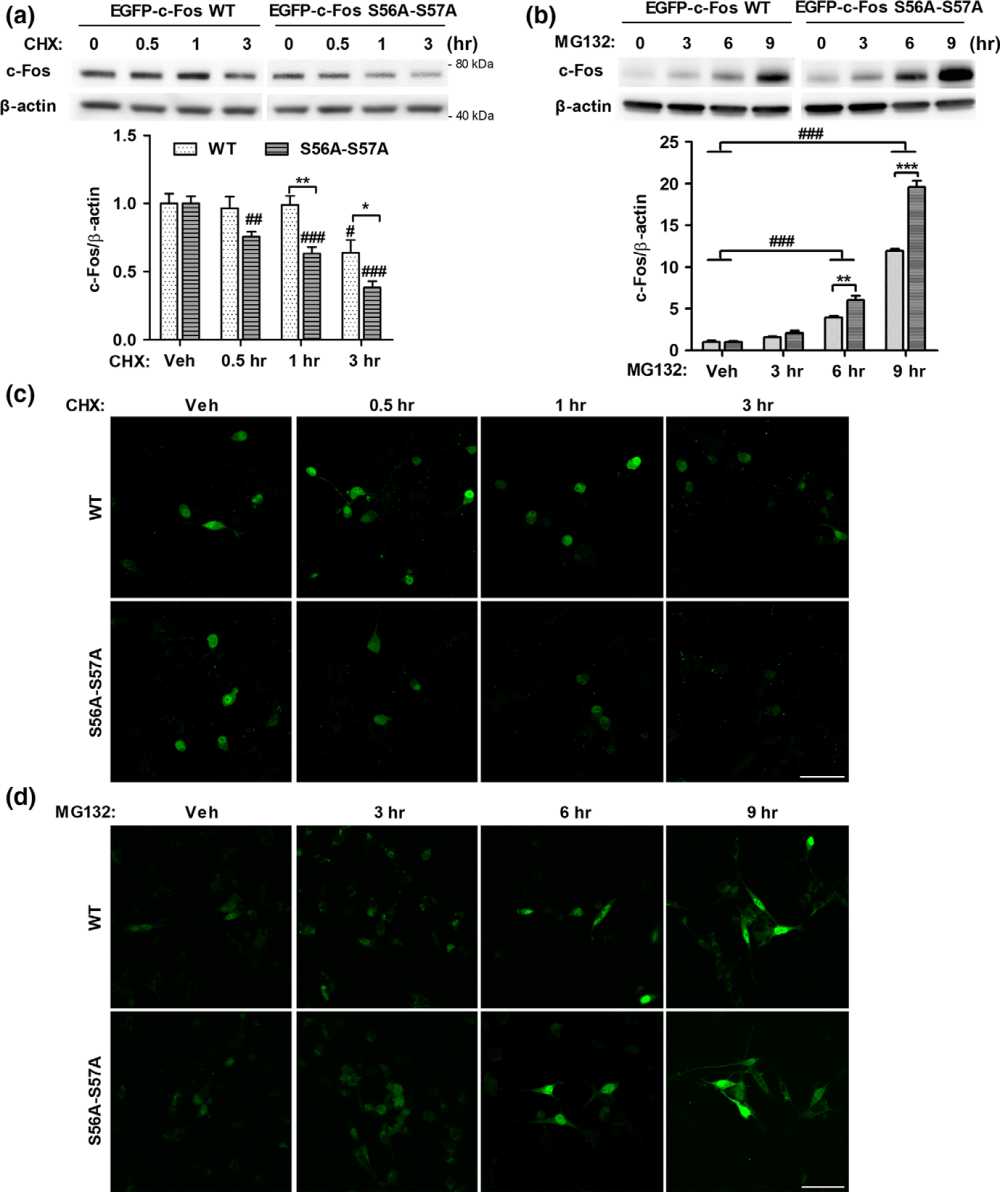
O‐GlcNAcylation of c‐Fos at S56 and S57 increases c‐Fos stability. (a, c) EGFP‐c‐Fos‐WT or EGFP‐c‐Fos‐S56A‐S57A transfected SH‐SY5Y were treated with cycloheximide (30 μg/mL) for indicated times. Representative immunoblot images and quantitative graph are shown in the upper panel and lower panel, respectively (a) (*n* = 4). Representative confocal images of EGFP signals are presented in (c). (b, d) EGFP‐c‐Fos‐WT or EGFP‐c‐Fos‐S56A‐S57A transfected SH‐SY5Y were treated with MG132 (5 μM) for indicated times. Representative immunoblot images and quantitative graph are shown in the upper panel and lower panel, respectively (b) (*n* = 5). Representative confocal images of EGFP signals are presented in (d). Data are shown as mean ± *SEM*. ^#^ *p* < 0.05, ^##^ *p* < 0.01, ^###^ *p* < 0.001 among EGFP‐c‐Fos‐WT or EGFP‐c‐Fos‐S56A‐S57A transfected group (one‐way ANOVA, Bonferroni posthoc test), *p < 0.05, **p < 0.01, ***p < 0.001 between EGFP‐c‐Fos‐WT and EGFP‐c‐Fos‐S56A‐S57A group (two‐way ANOVA, Bonferroni posthoc test). CHX: cycloheximide; S56A‐S57A: EGFP‐c‐Fos‐S56A‐S57A. Scale bar: 50 μm

Upon Aβ‐induced increase in c‐Fos O‐GlcNAcylation, the total levels of c‐Fos protein were also increased (Figure 1). Since N‐terminal region of c‐Fos is known to be a destabilizing region (Gomard et al., 2008), we hypothesized that O‐GlcNAcylation of c‐Fos contributes its stability. To determine whether O‐GlcNAcylation of c‐Fos at the sites S56 and S57 affects the stability of c‐Fos, EGFP‐c‐Fos‐WT, or EGFP‐c‐Fos‐S56A‐S57A transfected SH‐SY5Y cells were treated with cycloheximide, a translation inhibitor, or MG132, a proteasome inhibitor, in a time‐dependent manner (Figure 5). O‐GlcNAc silenced c‐Fos mutant was degraded starting from 0.5 hr, and c‐Fos‐WT from 3 hr after cycloheximide treatment (Figure 5a,c). The remaining c‐Fos‐WT and mutant levels were significantly different starting from 1 hr. These results suggest that c‐Fos‐WT has a higher stability compared to c‐Fos mutant, and that O‐GlcNAcylation at sites S56 and S57 increases c‐Fos stability. In addition, when proteasomal degradation was blocked, the levels of c‐Fos mutants were increased more than c‐Fos‐WT (Figure 5b,d) starting from 6 hr after MG132 treatment. This indicates that the amount of degraded c‐Fos mutant is increased compared to that of degraded c‐Fos‐WT at the same time point and that O‐GlcNAcylation of c‐Fos on S56 and S57 stabilizes c‐Fos against proteasomal degradation. Therefore, these experiments provide evidence that O‐GlcNAcylation of c‐Fos on sites S56 and S57 plays an important role in regulating its stability.

### 2.6 Aβ–induced c‐Fos O‐GlcNAcylation regulates transcriptional activity of c‐Fos and the expression of Bim

**Figure 6:**
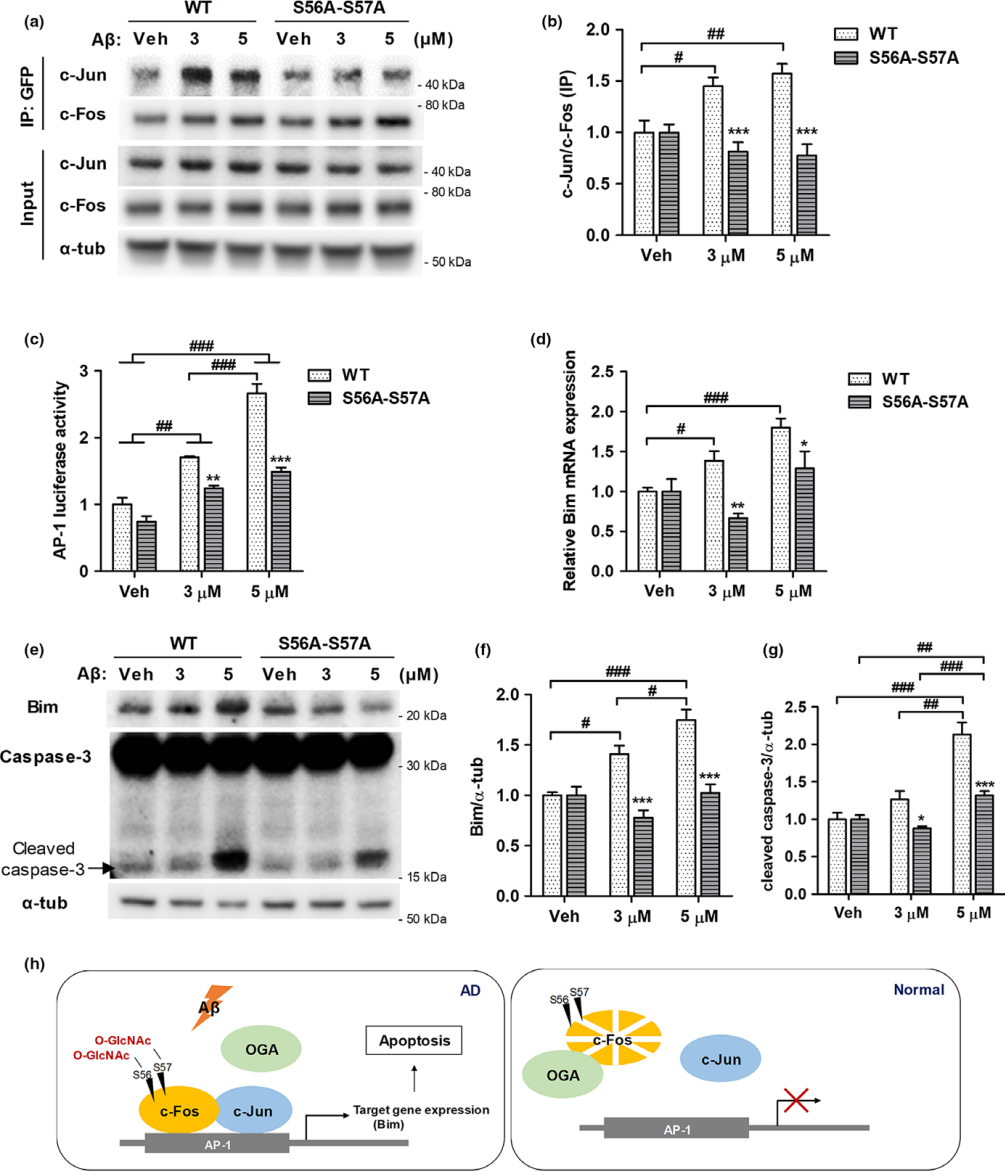
O‐GlcNAcylation of c‐Fos at S56 and S57 regulates the transcriptional activity of c‐Fos and the expression of Bim in the presence of Aβ. SH‐SY5Y (a, b, d–g) and HEK293T (c) cell lines were treated with Aβ (at doses indicated) for 24 hr. (a, b) The interaction between c‐Fos and c‐Jun was analyzed by performing immunoprecipitation. SH‐SY5Y cells were transfected with EGFP‐c‐Fos‐WT or EGFP‐c‐Fos‐S56A‐S57A. EGFP‐c‐Fos was immunoprecipitated using an anti‐GFP antibody and probed with c‐Jun. Representative immunoblot images are shown in (a) and quantitative graph in (b) (*n* = 5). (c) AP‐1 luciferase assay was performed in tag free‐c‐Fos‐WT or tag free‐c‐Fos‐S56A‐S57A in AP‐1 luciferase vector co‐transfected HEK293T cells (*n* = 4). (d) Relative Bim mRNA expression levels were measured in tag free‐c‐Fos‐WT or tag free‐c‐Fos‐S56A‐S57A transfected SH‐SY5Y cells (*n* = 5). (e–g) Relative expression levels of Bim and cleaved caspase‐3 were measured using western blotting in EGFP‐c‐Fos‐WT or EGFP‐c‐Fos‐S56A‐S57A transfected SH‐SY5Y cells (*n* = 5). Representative immunoblot images are shown in (e). Quantitative graphs showing the relative expression of Bim and cleaved caspase‐3 ((f) and (g), respectively). Data are shown as mean ± *SEM*. ^#^ *p* < 0.05, ^##^ *p* < 0.01, ^###^ *p* < 0.001 among c‐Fos‐WT or c‐Fos‐S56A‐S57A transfected groups (one‐way ANOVA, Bonferroni posthoc test), *p < 0.05, **p < 0.01, ***p < 0.001 between c‐Fos‐WT and c‐Fos‐S56A‐S57A groups (two‐way ANOVA, Bonferroni posthoc test). (h) Schematic diagram of the sites and the role of c‐Fos O‐GlcNAcylation in the presence of Aβ. O‐GlcNAc sites of c‐Fos are S56 and S57. c‐Fos O‐GlcNAcylation is increased by Aβ, which is mediated by a decreased interaction between c‐Fos and OGA. O‐GlcNAcylation of c‐Fos increases its stability and the interaction with c‐Jun, consequently elevating its transcriptional activity to induce the expression of Bim, an apoptotic protein, in the presence of Aβ. Therefore, O‐GlcNAcylation of c‐Fos promotes neuronal cell death in the presence of Aβ. α‐tub: α‐tubulin; IP: Immunoprecipitation; S56A‐S57A: c‐Fos‐S56A‐S57A; Veh: Vehicle; WT: c‐Fos‐WT

In the experiments illustrated in Figure 5, we observed that c‐Fos O‐GlcNAcylation plays a role in Aβ‐induced cell death, and we aimed to explore the mechanism underlying cell death caused by c‐Fos O‐GlcNAcylation. Since c‐Fos is a well‐known transcription factor that dimerizes with Jun family and binds AP‐1 DNA site (Hess et al., 2004), we hypothesized that the O‐GlcNAcylation of c‐Fos may regulate the dimerization of c‐Fos and c‐Jun, and consequently, the transcriptional activity of AP‐1 complex. As expected, we observed increased interaction of c‐Fos‐WT with c‐Jun compared to c‐Fos mutants in the presence of Aβ by immunoprecipitating EGFP‐c‐Fos (Figure 6a,b). Consistently, transcriptional activity of the AP‐1 complex, measured based on luciferase activity, was significantly higher in c‐Fos‐WT transfected cells compared to c‐Fos mutant transfected cells (Figure 6c). In addition, mRNA and protein levels of Bim, one of the AP‐1 target genes related to apoptosis (Shaulian & Karin, 2002), were dramatically increased by Aβ in c‐Fos‐WT transfected cells, while no changes occurred in c‐Fos mutant transfected cells (Figure 6d–f). Cleaved caspase‐3, a downstream target of Bim and the final effector protein of apoptosis, also showed a larger increase in c‐Fos‐WT transfected cells compared to c‐Fos mutant transfected cells in the presence of Aβ (Figure 6e,g). These results suggest that O‐GlcNAcylation of c‐Fos increases interactions between c‐Fos and c‐Jun, and, consequently, the transcriptional activity of AP‐1 resulting into an induction of Bim expression in the presence of Aβ. Taken together, we demonstrated that O‐GlcNAcylation of c‐Fos at sites S56 and S57 promotes cell death in the presence of Aβ by regulating its interaction with c‐Jun, AP‐1 transcriptional activity, and Bim expression, sequentially.

## 3 DISCUSSION

In this study, we revealed the novel sites and functions of O‐GlcNAcylation on c‐Fos in the presence of Aβ (Figure 6h). We identified, for the first time, that S56 and S57 are the O‐GlcNAc modified sites of c‐Fos. O‐GlcNAc cycling is known to be altered in the brains of patients with AD (Forster et al., 2014; Liu et al., 2009; Wang et al., 2017; Zhu et al., 2014), and altered c‐Fos O‐GlcNAcylation caused by Aβ may be one of the consequences of disrupted O‐GlcNAc cycling. In addition, we revealed that increased c‐Fos O‐GlcNAcylation by Aβ was mediated by a reduction in the interaction between OGA and c‐Fos. Increased c‐Fos O‐GlcNAcylation plays a role in stabilizing c‐Fos itself resulting in higher transcriptional activity of c‐Fos and c‐Jun complex leading to Bim expression. Bim is an apoptotic protein that translocates to the mitochondria and forms pores to release cytochrome c triggering caspase cascade under cytotoxic conditions (Vaid, 2011). Therefore, Aβ‐induced O‐GlcNAcylation at S56 and S57 sites of c‐Fos might promote cell death through an increase in Bim expression by the binding of c‐Fos/c‐Jun complex to the AP‐1 site.

Recently, OGA inhibition has been evoked as one of the therapeutic strategies for AD (Yuzwa & Vocadlo, 2014; Zhu et al., 2014). This was supported by several studies showing that AD‐like pathologies, such as memory impairment, hyperphosphorylated tau, neurofibrillary tangles, and Aβ plaques, were improved using OGA inhibitor for modulation of autophagy and the O‐GlcNAcylation of tau, and Nicastrin, one of γ‐secretase components (Kim et al., 2013; Graham et al., 2014; Yuzwa, Cheung, Okon, McIntosh, & Vocadlo, 2014; Yuzwa, Shan, et al., 2014; Zhu et al., 2018). However, several researches suggested that down regulation of OGA caused proteotoxicity resulted from accumulation of aggregate‐prone proteins such as huntingtin, Aβ and α‐synuclein (Wang et al., 2012; Wani et al., 2017). Moreover, elevating O‐GlcNAc by OGA inhibition or OGT overexpression induced mitochondrial dysfunction (Gawlowski et al., 2012; Tan, Villar, & E L, Lu J, Selfridge JE, Artigues A, Swerdlow RH, Slawson C, 2014). In addition, we demonstrated that increased O‐GlcNAcylation on c‐Fos elevated apoptosis in AD condition. These contradictory reports suggest that modulating O‐GlcNAc cycling rather than inhibiting OGA might be important therapeutic strategy for AD.

Liu et al. (2009) showed that the overall O‐GlcNAcylation was reduced in the brains of patients with AD (Liu et al., 2009). However, another report showed that the proteins of approximately 50 – 60 kDa and 25 kDa in size show increased O‐GlcNAcylation, while the proteins of over 70 kDa in size show reduced O‐GlcNAcylation in the brains of patients with AD (Forster et al., 2014). In addition, a recent study on proteomic analysis using the brains of AD patients showed increased abundance of O‐GlcNAc peptides (Wang et al., 2017). Another proteomic study using 3xTg‐AD mice reported that some of the O‐GlcNAcylated proteins present only in 3xTg‐AD mice (Alfaro et al., 2012). These data suggested that O‐GlcNAc cycling in AD was altered rather than simply reduced. Thus, increased O‐GlcNAcylation on c‐Fos may be a result of altered O‐GlcNAc cycling in AD.

The regulation of O‐GlcNAc cycling seems to be complicated and the levels and activities of O‐GlcNAc modifying enzymes seemed not to be changed in AD (Wang et al., 2017). Nevertheless, there are several possible regulatory mechanisms. First, the interactions between O‐GlcNAc modifying enzymes (OGT or OGA) and their substrates might be altered. It has been reported that the interaction between OGT and its substrate (ATP synthase 5A) is altered by hindering inhibition of Aβ (Cha et al., 2015). We also observed that the interaction between OGA and c‐Fos was reduced. Second, there were reports that several kinases regulate substrate specificities of OGT and OGA (Nagel & Ball, 2014). For example, p38 MAPK affects O‐GlcNAcylation of neurofilament H in neuroblastoma cells by interacting with OGT and not affecting phosphorylation (Cheung & Hart, 2008). Although these phenomena have not been tested in AD condition, it is known that kinases are dysregulated in AD (Dolan & Johnson, 2010; Perluigi, Barone, Domenico, & Butterfield, 2016). Thus, kinases might be involved in altered O‐GlcNAc cycling such as disrupted OGA and c‐Fos interaction in AD. Third, phosphorylation might affect O‐GlcNAcylation by competitive or synergistic manner (Bond & Hanover, 2015). A recent study revealed that the phosphorylation/O‐GlcNAcylation interplay motif, (pS/pT)P(V/A/T)(gS/gT), and around O‐GlcNAcylation sites of tau resemble to this motif (Leney, Atmioui, Wu, Ovaa, & Heck, 2017). However, S56 and S57 containing peptide of c‐Fos is different from that motif, and there has been no report about phosphorylation around or on S56 and S57 of c‐Fos so far. Thus, c‐Fos O‐GlcNAcylation may be regulated differently from tau. Although we described possible O‐GlcNAc regulatory mechanisms, the precise regulatory mechanism of decreased OGA binding to c‐Fos in response to Aβ and altered O‐GlcNAc cycling in AD needs to be further studied. In addition, it requires further investigation whether O‐GlcNAcylation on substrates affect the ability of interaction between O‐GlcNAc modifying enzymes and substrates or not.

We revealed that c‐Fos O‐GlcNAcylation at S56 and S57 can improve its stability. It has been reported that N‐terminal region of c‐Fos is a destabilizer (Ferrara et al., 2003; Gomard et al., 2008), although the specific sequence of this region is not clear. It is also known that phosphorylation at S32 of c‐Fos by ERK5 inhibits its degradation by the proteasome (Sasaki et al., 2006). Thus, a potential reason that c‐Fos O‐GlcNAcylation can regulate its stability may be that S56 and S57 sites of c‐Fos might be present within N‐terminal destabilizer. In contrast, O‐GlcNAcylation at S56 and S57 might affect the phosphorylation on S32 of c‐Fos. We also demonstrated that the interaction between c‐Fos and c‐Jun was increased by c‐Fos O‐GlcNAcylation in the presence of Aβ. Although S56 and S57 are located far from DNA binding‐ (amino acids 139–160) or leucine zipper‐domains (amino acids 165–193) (Eferl & Wagner, 2003), O‐GlcNAcylation at S56 and S57 might affect the cross‐talk with transcriptional co‐factors, such as CBP, CRTC1, BAF complex, by altering the three‐dimensional configuration of these components (Bannister & Kouzarides, 1995; Canettieri et al., 2009; Vierbuchen et al., 2017). However, the precise underlying mechanisms need further investigation.

Several AP‐1 target genes under cytotoxic stimuli have been described, such as Fas ligand (FasL), Fas, and Bim (Chen et al., 2015; Shaulian & Karin, 2002; Whitfield et al., 2001). We tested the levels of these proteins in our experiments, and observed that those of FasL and Fas were not altered by Aβ (Supporting Information Figure S5). The expression of various AP‐1 target genes depends on specific stimuli, thus, it seems that Aβ can induce only Bim among apoptotic AP‐1 target genes in SH‐SY5Y cells. Our study focused on c‐Fos and revealed that post‐translational modification of c‐Fos can regulate the transcriptional activity of AP‐1 complex. In terms of cell death, there were other cell death‐related proteins which was altered O‐GlcNAcylation in the brains of AD patients such as MECP2, NTRK2, LMNA. The roles of altered O‐GlcNAcylation of these proteins need to be investigated to understanding the link between cell death and disrupted glucose metabolism.

Dysregulated glucose metabolism is involved in several human diseases such as diabetes mellitus and AD (Bond & Hanover, 2015). O‐GlcNAc cycling, altered in the brains of patients with AD, reflects dysregulated glucose metabolism (Schubert, 2005; Zhu et al., 2014). Here, we demonstrated altered c‐Fos O‐GlcNAcylation in AD and revealed that the function of c‐Fos O‐GlcNAcylation is to increase the stability of c‐Fos, stimulating its interaction with c‐Jun and the transcriptional activity followed by an induction of Bim, an apoptotic gene, expression therefore directly promoting cell death in the presence of Aβ. These results provide an insight into the effects of dysregulated O‐GlcNAc cycling in AD pathophysiology. Therefore, modulating O‐GlcNAc cycling rather than inhibiting OGA might be a potential therapeutic strategy for AD.

## 4 EXPERIMENTAL PROCEDURES

### 4.1 Animals

Eight‐month‐old 5xFAD mice (Tg6799; B6SJL‐Tg (APPSwFlLon, PSEN* M146L*L286V) 6799Vas/J, stock number 006554, Jackson Labs, Bar Harbor, ME, USA) overexpressing human amyloid precursor protein 695 with three mutations (Swedish, Florida, and London) and human presenilin 1 with two mutations (M146L and L286V) under transcriptional control of the murine Thy‐1 promoter and wild‐type littermate (B6/SJL) were used for brain tissue analysis. Animal experiments were performed in accordance with the Principle of Laboratory Animal Care (NIH publication No. 85‐23, revised 1985) and the Animal Care and Use Guidelines of Seoul National University, Seoul, Korea. All experimental protocols were approved by Institutional Animal Care and Use Committee (IACUC) at Seoul National University.

### 4.2 Primary neuronal culture

Primary cortical neuronal cultures were prepared as previously described (Jung, An, Hong, Kim, & Mook‐Jung, 2012). In brief, brain tissue of Sprague‐Dawley rat embryos (E18) was dissected (KOATECH, Korea), the brains were trypsinized in Hank's Balanced Salt Solution (HBSS; WelGENE, Korea), and dissociation was performed in NeuroBasal medium (Gibco, USA) supplemented with B27 (Gibco, USA) and penicillin/streptomycin (Sigma, USA). Dissociated neurons were plated on poly‐D‐lysine (Sigma, USA) coated dishes. Half of the culture medium was replaced with fresh medium every three days.

### 4.3 Cell culture and transfection

SH‐SY5Y and HEK293T cell lines were grown in Dulbecco's modified Eagles medium (DMEM; HyClone, USA) supplemented with 10% fetal bovine serum (Hyclone, USA) and 1% penicillin/streptomycin (Sigma, USA) at 37°C under 5% CO_2_. For transfection, constructs or siRNA were mixed with Lipofectamine^®^ or Lipofectamine LTX and Plus reagent (Invitrogen, USA) in Opti‐MEM (Gibco, USA) for SH‐SY5SY or HEK293T cells, respectively.

### 4.4 DNA constructs, siRNA, reagents

EGFP‐tagged c‐Fos constructs (EGFP‐c‐Fos‐WT and point‐mutated EGFP‐c‐Fos constructs) and two tag free c‐Fos constructs (c‐Fos‐WT and c‐Fos‐S56A‐S57A) were used. Tag‐free c‐Fos‐WT plasmid was purchased from OriGene (USA). c‐Fos‐S56A‐S57A plasmid was generated by introducing a point mutation into c‐Fos‐WT plasmid using a site‐directed mutagenesis kit (Enzynomics, Korea) according to manufacturer's instructions. EGFP‐tagged c‐Fos constructs were generated using Gateway cloning technology (Invitrogen, USA). AP‐1‐luc plasmid, containing luciferase gene under transcriptional control of AP‐1 promoter, was purchased from Promega (USA). OGT siRNA was predesigned and synthesized by Bioneer (Korea). Thiamet G and β‐hexosaminidase were purchased from Sigma (USA) and Aβ_1‐42_ peptides (American peptide and Bachem, USA) were used.

### 4.5 Preparation of Aβ

Aβ_1‐42_ peptide (American peptide, USA and Bachem, Switzerland) was prepared as previously described (Byun et al., 2015). In brief, Aβ_1‐42_ peptide was dissolved in 1,1,1,3,3,3‐hexafluoro‐2‐propanol (Sigma, USA) and lyophilized in a Speedvac (Labconco, USA). Dry peptide was dissolved in anhydrous dimethyl sulfoxide (Sigma, USA) at a final concentration of 1 mM and diluted in DMEM or cell culture medium. During treatment in cell culture, most of Aβ consisted predominantly of oligomers and a few monomers (Kim et al., 2012).

### 4.6 Wheat‐Germ‐Agglutinin‐Agarose‐Pull Down, Immunoprecipitation And Western Blotting

For WGA‐pull down assay, cells or brain tissues were lysed in RIPA buffer (iNtRON Biotechonology, Korea) containing a protease inhibitor cocktail, phenyl‐methylsulfonyl fluoride (PMSF; Sigma, USA), and an OGA inhibitor. O‐GlcNAcylated proteins were pulled down using WGA bounded agarose beads (Vector, USA) at 4°C overnight. For immunoprecipitation of EGFP‐tagged c‐Fos, cells were lysed in 1% Triton X‐100 in TBS buffer (50 mM Tris HCl, 150 mM NaCl, pH 7.4) containing a protease inhibitor cocktail, PMSF, and an OGA inhibitor. Lysates were incubated with an anti‐GFP antibody (Abcam, ab1218, USA) at 4°C overnight and with protein A/G agarose beads (SantaCruz, USA) at 4°C for 3 hr. In both cases, beads were washed with each lysis buffer three times and eluted with SDS‐PAGE sample buffer by boiling at 95°C for 5 min and using a vortex. Both boiled elutes and equal amounts of input samples were separated via SDS‐PAGE and transferred to polyvinylidene difluoride (PVDF) membranes. Membranes were blocked using 5% skim milk (Bioworld, USA) and probed with antibodies against indicated proteins. The protocol for western blotting has been also described in a previous report.(Kim et al., 2012) The antibodies used were anti‐c‐Fos (CST4384 and CST2250, CST technologies, USA and sc‐52, Santacruz, USA), anti‐c‐Jun (CST9165), anti‐Bim (CST2933), and anti‐Caspase‐3 (CST9662), anti‐ O‐GlcNAc (CTD110.6, MMS‐248R, Covance, USA), anti‐GFP (ab1218, Abcam, USA for immunoprecipitation and sc‐9996, SantaCruz, USA for immunoblotting), anti‐OGA (SAB4200267, Sigma, USA), anti‐OGT (O6264, Sigma) and anti‐β‐actin (A5441, Sigma), anti‐α‐tubulin (05‐829, Millipore, Germany).

### 4.7 Fluorescence imaging

For analyzing OGA and c‐Fos interaction, we performed immunocytochemistry as previously described (Kim et al., 2012). Briefly, EGFP‐c‐Fos‐WT transfected cells were plated on coverslips and treated by Aβ. After fixed by 4% paraformaldehyde (BIOSESANG, Inc., Korea), cells were incubated with anti‐OGA (Sigma, USA) and GFP (OSE00001G, Osenses, Australia) antibodies, which were diluted in 0.5% Tx‐100, 1% goat serum in PBS, overnight at 4°C. After labeling with fluorescent‐labeled secondary antibodies (Invitrogen, USA) for 1 hr at room temperature, cells were imaged by SIM (Nikon N‐SIM, Nikon Instruments, Inc., Japan). Images were processed and analyzed by NIS‐E software (Nikon Instruments, Inc., Japan). For imaging c‐Fos level, native EGFP signals were imaged by confocal microscopy (Olympus FV10i; Olympus, Japan and LSM710; Carl Zeiss, Germany) in living cells which were transfected by EGFP‐c‐Fos‐WT and treated by CHX or MG132.

### 4.8 TUNEL assay

For measuring cell death, EGFP‐c‐Fos transfected cells were fixed by 4% paraformaldehyde after drug treatment, and permeabilized by 0.1% Tx‐100 in PBS. After incubated with TUNEL reaction mixture (TMR Red; Roche, Germany) for 1 hr at 37°C in dark, cells were imaged by fluorescent microscopy (EVOS FL Auto2; Thermo Fisher Scientific, USA). Images were processed and analyzed by Celleste software (Thermo Fisher Scientific, USA).

### 4.9 Calcein‐AM assay

For measuring cell viability, cells were incubated with 1 μM of Calcein‐AM (Invitrogen, USA) in DMEM for 1 hr at 37°C. After changing the medium to PBS, fluorescent signals were measured using luminometer (excitation at 490 nm and emission at 520 nm).

### 4.10 MTS assay

For measuring cell viability, cells were treated with MTS solution (Promega, USA) in cell culture medium. After 1 hr incubation at 37°C, signals were measured using a spectrophotometer at 490 nm.

### 4.11 Luciferase assay

The cells transfected with AP‐1‐luc plasmid were lysed in passive lysis buffer (Promega, USA). Equal amounts of cell lysates were mixed with a luciferase assay reagent (Promega, USA), and then the measurement was performed using luminometer. These processes were performed according to manufacturer's instructions (Promega, USA).

### 4.12 Statistical analysis

Data were analyzed using unpaired *t*‐tests or one‐way analysis of variance (ANOVA) or two‐way ANOVA with Bonferroni posthoc tests for multiple comparisons. *p* values <0.05 were considered as statistically significant. All data are shown as mean ± *SEM*.

## CONFLICT OF INTEREST

The authors declare no conflict of interest.

## AUTHOR CONTRIBUTIONS

HC, CK, and IM‐J designed the research. HC, HS, M‐YC, and HJK performed the experiments and analyzed data. HJC and SMS served intellectual contributions about experiments and analyzing data. HC and IM‐J wrote the manuscript. IM‐J supervised the entire project.

## References

[acel12872-bib-0001] Alfaro, J. F. , Gong, C. X. , Monroe, M. E. , Aldrich, J. T. , Clauss, T. R. , Purvine, S. O. , … Smith, R. D. (2012). Tandem mass spectrometry identifies many mouse brain O‐GlcNAcylated proteins including EGF domain‐specific O‐GlcNAc transferase targets. Proceedings of the National Academy of Sciences of the United States of America, 109, 7280–7285. 10.1073/pnas.1200425109 22517741 PMC3358849

[acel12872-bib-0002] Bannister, A. J. , & Kouzarides, T. (1995). CBP‐induced stimulation of c‐Fos activity is abrogated by E1A. EMBO Journal, 14, 4758–4762. 7588605 10.1002/j.1460-2075.1995.tb00157.x PMC394573

[acel12872-bib-0003] Bond, M. R. , & Hanover, J. A. (2015). A little sugar goes a long way: The cell biology of O‐GlcNAc. Journal of Cell Biology, 208, 869–880. 25825515 10.1083/jcb.201501101 PMC4384737

[acel12872-bib-0004] Byun, J. , Son, S. M. , Cha, M. Y. , Shong, M. , Hwang, Y. J. , Kim, Y. , … Mook‐Jung, I. (2015). CR4‐interacting factor 1 is a key regulator in Abeta‐induced mitochondrial disruption and pathogenesis of Alzheimer’s disease. Cell Death and Differentiation, 22, 959–973. 25361083 10.1038/cdd.2014.184 PMC4423180

[acel12872-bib-0005] Canettieri, G. , Coni, S. , Della Guardia, M. , Nocerino, V. , Antonucci, L. , Di Magno, L. , … Gulino, A. (2009). The coactivator CRTC1 promotes cell proliferation and transformation via AP‐1. Proceedings of the National Academy of Sciences of the United States of America, 106, 1445–1450. 10.1073/pnas.0808749106 19164581 PMC2635810

[acel12872-bib-0006] Cha, M. Y. , Cho, H. J. , Kim, C. , Jung, Y. O. , Kang, M. J. , Murray, M. E. , … Mook‐Jung, I. (2015). Mitochondrial ATP synthase activity is impaired by suppressed O‐GlcNAcylation in Alzheimer’s disease. Human Molecular Genetics, 24, 6492–6504. 26358770 10.1093/hmg/ddv358 PMC5007609

[acel12872-bib-0007] Chen, X. , Shen, J. , Wang, Y. , Chen, X. , Yu, S. , Shi, H. , & Huo, K. (2015). Up‐regulation of c‐Fos associated with neuronal apoptosis following intracerebral hemorrhage. Cellular and Molecular Neurobiology, 35, 363–376. 10.1007/s10571-014-0132-z 25354492 PMC11486182

[acel12872-bib-0008] Cheung, W. D. , & Hart, G. W. (2008). AMP‐activated protein kinase and p38 MAPK activate O‐GlcNAcylation of neuronal proteins during glucose deprivation. Journal of Biological Chemistry, 283, 13009–13020. 18353774 10.1074/jbc.M801222200 PMC2435304

[acel12872-bib-0009] Dolan, P. J. , & Johnson, G. V. (2010). The role of tau kinases in Alzheimer’s disease. Current Opinion in Drug Discovery & Development, 13, 595–603. 20812151 PMC2941661

[acel12872-bib-0010] Eferl, R. , & Wagner, E. F. (2003). AP‐1: A double‐edged sword in tumorigenesis. Nature Reviews Cancer, 3, 859–868. 10.1038/nrc1209 14668816

[acel12872-bib-0011] Fernandez, M. , Pirondi, S. , Antonelli, T. , Ferraro, L. , Giardino, L. , & Calza, L. (2005). Role of c‐Fos protein on glutamate toxicity in primary neural hippocampal cells. Journal of Neuroscience Research, 82, 115–125. 10.1002/jnr.20608 16075465

[acel12872-bib-0012] Ferrara, P. , Andermarcher, E. , Bossis, G. , Acquaviva, C. , Brockly, F. , Jariel‐Encontre, I. , & Piechaczyk, M. (2003). The structural determinants responsible for c‐Fos protein proteasomal degradation differ according to the conditions of expression. Oncogene, 22, 1461–1474. 10.1038/sj.onc.1206266 12629509

[acel12872-bib-0013] Forster, S. , Welleford, A. S. , Triplett, J. C. , Sultana, R. , Schmitz, B. , & Butterfield, D. A. (2014). Increased O‐GlcNAc levels correlate with decreased O‐GlcNAcase levels in Alzheimer disease brain. Biochimica et Biophysica Acta, 1842, 1333–1339. 10.1016/j.bbadis.2014.05.014 24859566 PMC4140188

[acel12872-bib-0014] Gawlowski, T. , Suarez, J. , Scott, B. , Torres‐Gonzalez, M. , Wang, H. , Schwappacher, R. , … Dillmann, W. (2012). Modulation of dynamin‐related protein 1 (DRP1) function by increased O‐linked‐beta‐N‐acetylglucosamine modification (O‐GlcNAc) in cardiac myocytes. Journal of Biological Chemistry, 287, 30024–30034. 22745122 10.1074/jbc.M112.390682 PMC3436129

[acel12872-bib-0015] Gillardon, F. , Skutella, T. , Uhlmann, E. , Holsboer, F. , Zimmermann, M. , & Behl, C. (1996). Activation of c‐Fos contributes to amyloid beta‐peptide‐induced neurotoxicity. Brain Research, 706, 169–172. 8720507 10.1016/0006-8993(95)01332-6

[acel12872-bib-0016] Gomard, T. , Jariel‐Encontre, I. , Basbous, J. , Bossis, G. , Moquet‐Torcy, G. , & Piechaczyk, M. (2008). Fos family protein degradation by the proteasome. Biochemical Society Transactions, 36, 858–863. 18793151 10.1042/BST0360858

[acel12872-bib-0017] Graham, D. L. , Gray, A. J. , Joyce, J. A. , Yu, D. , O’Moore, J. , Carlson, G. A. , … Hering, H. (2014). Increased O‐GlcNAcylation reduces pathological tau without affecting its normal phosphorylation in a mouse model of tauopathy. Neuropharmacology, 79, 307–313. 10.1016/j.neuropharm.2013.11.025 24326295

[acel12872-bib-0018] Hess, J. , Angel, P. , & Schorpp‐Kistner, M. (2004). AP‐1 subunits: Quarrel and harmony among siblings. Journal of Cell Science, 117, 5965–5973. 10.1242/jcs.01589 15564374

[acel12872-bib-0019] Jung, E. S. , An, K. , Hong, H. S. , Kim, J. H. , & Mook‐Jung, I. (2012). Astrocyte‐originated ATP protects Abeta(1‐42)‐induced impairment of synaptic plasticity. Journal of Neuroscience, 32, 3081–3087. 22378880 10.1523/JNEUROSCI.6357-11.2012 PMC6622014

[acel12872-bib-0020] Kim, C. , Choi, H. , Jung, E. S. , Lee, W. , Oh, S. , Jeon, N. L. , & Mook‐Jung, I. (2012). HDAC6 inhibitor blocks amyloid beta‐induced impairment of mitochondrial transport in hippocampal neurons. PLoS One, 7, e42983 10.1371/journal.pone.0042983 22937007 PMC3425572

[acel12872-bib-0021] Kim, C. , Nam, D. W. , Park, S. Y. , Song, H. , Hong, H. S. , Boo, J. H. , … Mook‐Jung, I. (2013). O‐linked beta‐N‐acetylglucosaminidase inhibitor attenuates beta‐amyloid plaque and rescues memory impairment. Neurobiology of Aging, 34, 275–285. 22503002 10.1016/j.neurobiolaging.2012.03.001

[acel12872-bib-0022] Kumar, A. , Singh, A. , & Ekavali,, (2015). A review on Alzheimer’s disease pathophysiology and its management: An update. Pharmacological Reports, 67, 195–203. 10.1016/j.pharep.2014.09.004 25712639

[acel12872-bib-0023] Leney, A. C. , El Atmioui, D. , Wu, W. , Ovaa, H. , & Heck, A. J. R. (2017). Elucidating crosstalk mechanisms between phosphorylation and O‐GlcNAcylation. Proceedings of the National Academy of Sciences of the United States of America, 114, E7255–E7261. 10.1073/pnas.1620529114 28808029 PMC5584407

[acel12872-bib-0024] Liu, F. , Shi, J. , Tanimukai, H. , Gu, J. , Gu, J. , Grundke‐Iqbal, I. , … Gong, C. X. (2009). Reduced O‐GlcNAcylation links lower brain glucose metabolism and tau pathology in Alzheimer’s disease. Brain, 132, 1820–1832. 10.1093/brain/awp099 19451179 PMC2702834

[acel12872-bib-0025] Marcus, D. L. , Strafaci, J. A. , Miller, D. C. , Masia, S. , Thomas, C. G. , Rosman, J. , … Freedman, M. L. (1998). Quantitative neuronal c‐fos and c‐jun expression in Alzheimer’s disease. Neurobiology of Aging, 19, 393–400. 9880041 10.1016/s0197-4580(98)00077-3

[acel12872-bib-0026] Nagel, A. K. , & Ball, L. E. (2014). O‐GlcNAc transferase and O‐GlcNAcase: Achieving target substrate specificity. Amino Acids, 46, 2305–2316. 25173736 10.1007/s00726-014-1827-7 PMC4584397

[acel12872-bib-0027] Perluigi, M. , Barone, E. , Di Domenico, F. , & Butterfield, D. A. (2016). Aberrant protein phosphorylation in Alzheimer disease brain disturbs pro‐survival and cell death pathways. Biochimica et Biophysica Acta, 1862, 1871–1882. 10.1016/j.bbadis.2016.07.005 27425034

[acel12872-bib-0028] Querfurth, H. W. , & LaFerla, F. M. (2010). Alzheimer’s disease. New England Journal of Medicine, 362, 329–344. 20107219 10.1056/NEJMra0909142

[acel12872-bib-0029] Sasaki, T. , Kojima, H. , Kishimoto, R. , Ikeda, A. , Kunimoto, H. , & Nakajima, K. (2006). Spatiotemporal regulation of c‐Fos by ERK5 and the E3 ubiquitin ligase UBR1, and its biological role. Molecular Cell, 24, 63–75. 10.1016/j.molcel.2006.08.005 17018293

[acel12872-bib-0030] Schubert, D. (2005). Glucose metabolism and Alzheimer’s disease. Ageing Research Reviews, 4, 240–257. 10.1016/j.arr.2005.02.003 15950548

[acel12872-bib-0031] Shaulian, E. , & Karin, M. (2002). AP‐1 as a regulator of cell life and death. Nature Cell Biology, 4, E131–E136. 10.1038/ncb0502-e131 11988758

[acel12872-bib-0032] Tai, H. C. , Khidekel, N. , Ficarro, S. B. , Peters, E. C. , & Hsieh‐Wilson, L. C. (2004). Parallel identification of O‐GlcNAc‐modified proteins from cell lysates. Journal of the American Chemical Society, 126, 10500–10501. 15327282 10.1021/ja047872b

[acel12872-bib-0033] Tan, E. P. , Villar, M. T. , E, L. , Lu, J. , Selfridge, J. E. , Artigues, A. , Swerdlow, R. H. , & Slawson, C. (2014). Altering O‐linked beta‐N‐acetylglucosamine cycling disrupts mitochondrial function. Journal of Biological Chemistry, 289, 14719–14730. 10.1074/jbc.M113.525790 24713701 PMC4031527

[acel12872-bib-0034] Vaid, A. (2011). Nilotinib as first‐line therapy for chronic myeloid leukemia. Indian Journal of Cancer, 48, 438–445. 10.4103/0019-509X.92274 22293257

[acel12872-bib-0035] Vierbuchen, T. , Ling, E. , Cowley, C. J. , Couch, C. H. , Wang, X. , Harmin, D. A. , … Greenberg, M. E. (2017). AP‐1 transcription factors and the BAF complex mediate signal‐dependent enhancer selection. Molecular Cell, 68(1067–1082), e1012 10.1016/j.molcel.2017.11.026 PMC5744881 29272704

[acel12872-bib-0036] Wang, P. , Lazarus, B. D. , Forsythe, M. E. , Love, D. C. , Krause, M. W. , & Hanover, J. A. (2012). O‐GlcNAc cycling mutants modulate proteotoxicity in *Caenorhabditis elegans* models of human neurodegenerative diseases. Proceedings of the National Academy of Sciences of the United States of America, 109, 17669–17674. 10.1073/pnas.1205748109 22988095 PMC3491483

[acel12872-bib-0037] Wang, S. , Yang, F. , Petyuk, V. A. , Shukla, A. K. , Monroe, M. E. , Gritsenko, M. A. , … Liu, T. (2017). Quantitative proteomics identifies altered O‐GlcNAcylation of structural, synaptic and memory‐associated proteins in Alzheimer’s disease. The Journal of Pathology, 243, 78–88. 10.1002/path.4929 28657654 PMC5647145

[acel12872-bib-0038] Wani, W. Y. , Ouyang, X. , Benavides, G. A. , Redmann, M. , Cofield, S. S. , Shacka, J. J. , … Zhang, J. (2017). O‐GlcNAc regulation of autophagy and alpha‐synuclein homeostasis; implications for Parkinson’s disease. Mol Brain., 10, 32. 28724388 10.1186/s13041-017-0311-1 PMC5517830

[acel12872-bib-0039] Whitfield, J. , Neame, S. J. , Paquet, L. , Bernard, O. , & Ham, J. (2001). Dominant‐negative c‐Jun promotes neuronal survival by reducing BIM expression and inhibiting mitochondrial cytochrome c release. Neuron, 29, 629–643. 10.1016/S0896-6273(01)00239-2 11301023

[acel12872-bib-0040] Yuzwa, S. A. , Cheung, A. H. , Okon, M. , McIntosh, L. P. , & Vocadlo, D. J. (2014). O‐GlcNAc modification of tau directly inhibits its aggregation without perturbing the conformational properties of tau monomers. Journal of Molecular Biology, 426, 1736–1752. 24444746 10.1016/j.jmb.2014.01.004

[acel12872-bib-0041] Yuzwa, S. A. , Shan, X. , Jones, B. A. , Zhao, G. , Woodward, M. L. , Li, X. , … Vocadlo, D. J. (2014). Pharmacological inhibition of O‐GlcNAcase (OGA) prevents cognitive decline and amyloid plaque formation in bigenic tau/APP mutant mice. Molecular Neurodegeneration, 9, 42. 25344697 10.1186/1750-1326-9-42 PMC4232697

[acel12872-bib-0042] Yuzwa, S. A. , & Vocadlo, D. J. (2014). O‐GlcNAc and neurodegeneration: Biochemical mechanisms and potential roles in Alzheimer’s disease and beyond. Chemical Society Reviews, 43, 6839–6858. 24759912 10.1039/c4cs00038b

[acel12872-bib-0043] Zhang, J. , Zhang, D. , McQuade, J. S. , Behbehani, M. , Tsien, J. Z. , & Xu, M. (2002). c‐fos regulates neuronal excitability and survival. Nature Genetics, 30, 416–420. 10.1038/ng859 11925568

[acel12872-bib-0044] Zhu, Y. , Shan, X. , Safarpour, F. , Erro Go, N. , Li, N. , Shan, A. , … Vocadlo, D. J. (2018). Pharmacological inhibition of O‐GlcNAcase enhances autophagy in brain through an mTOR‐independent pathway. ACS Chemical Neuroscience, 9(6), 1366–1379. 10.1021/acschemneuro.8b00015 29460617

[acel12872-bib-0045] Zhu, Y. , Shan, X. , Yuzwa, S. A. , & Vocadlo, D. J. (2014). The emerging link between O‐GlcNAc and Alzheimer disease. Journal of Biological Chemistry, 289, 34472–34481. 25336656 10.1074/jbc.R114.601351 PMC4263855

