# Supplementary material for: Amyloid β‐induced elevation of O‐GlcNAcylated c‐Fos promotes neuronal cell death
Source: Aging Cell. 2018 Dec 4;18(1):e12872. doi: 10.1111/acel.12872 (PMC6351842; doi:10.1111/acel.12872)
Supplement: Supplementary file 1 [file ACEL-18-e12872-s001.pdf]

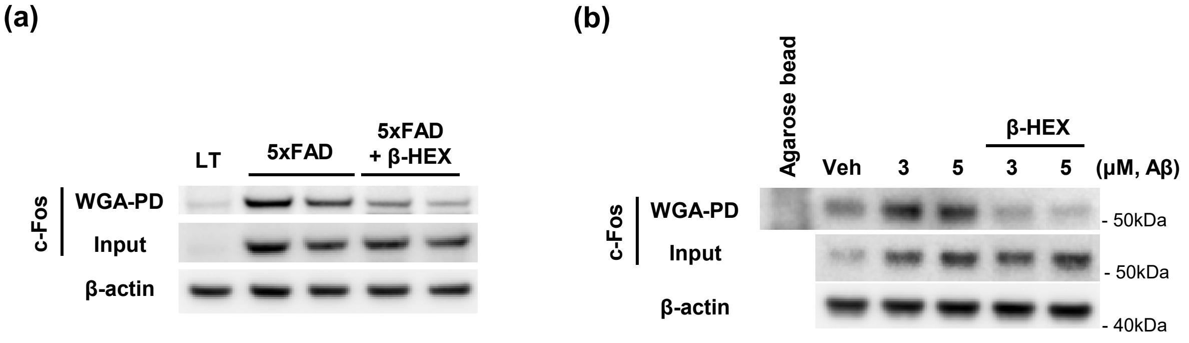

Figure S1. c-Fos is O-GlcNAcylated in the presence of Aβ

β-HEX assay was performed using the brains of 5xFAD mice and SH-SY5Y cells. Total tissue or cell lysates and WGA-pulled down-O-GlcNAcylated proteins were incubated with β-HEX overnight at 37 °C. (a) Representative immunoblot images of β-HEX assay in 5xFAD mice. (b) Representative immunoblot images of β-HEX assay in Aβ treated SH-SY5Y cell lines. WGA-PD: wheat-germ-agglutinin pull-down, LT: wild-type littermate, Veh: Vehicle, β-HEX: β-hexosaminidase.

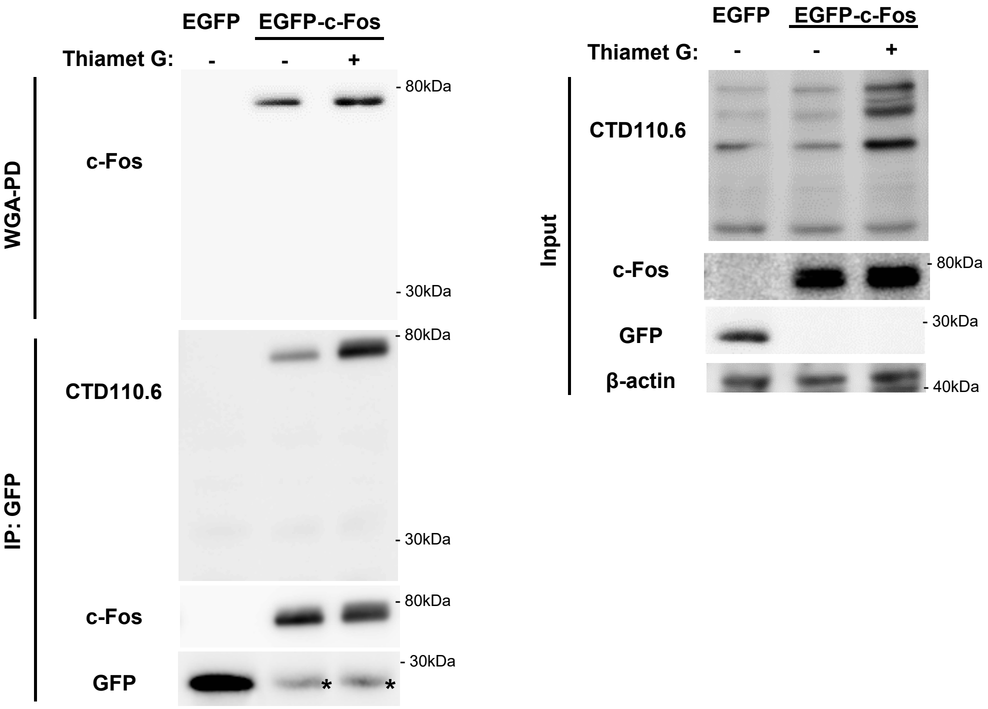

Figure S2. Confirmation of c-Fos O-GlcNAcylation in HEK293T cell line
EGFP or EGFP-c-Fos transfected HEK293T cells were treated with or without thiamet G (1 μM) for 24 h. EGFP or EGFP-c-Fos was WGA-pulled down or immunoprecipitated using an anti-GFP antibody and probed with indicated antibodies. c-Fos is O-GlcNAcylated in HEK293T cell lines. The stars may light-chains of anti-GFP antibodies. WGA-PD: wheat-germ-agglutinin pull-down, IP: Immunoprecipitation.

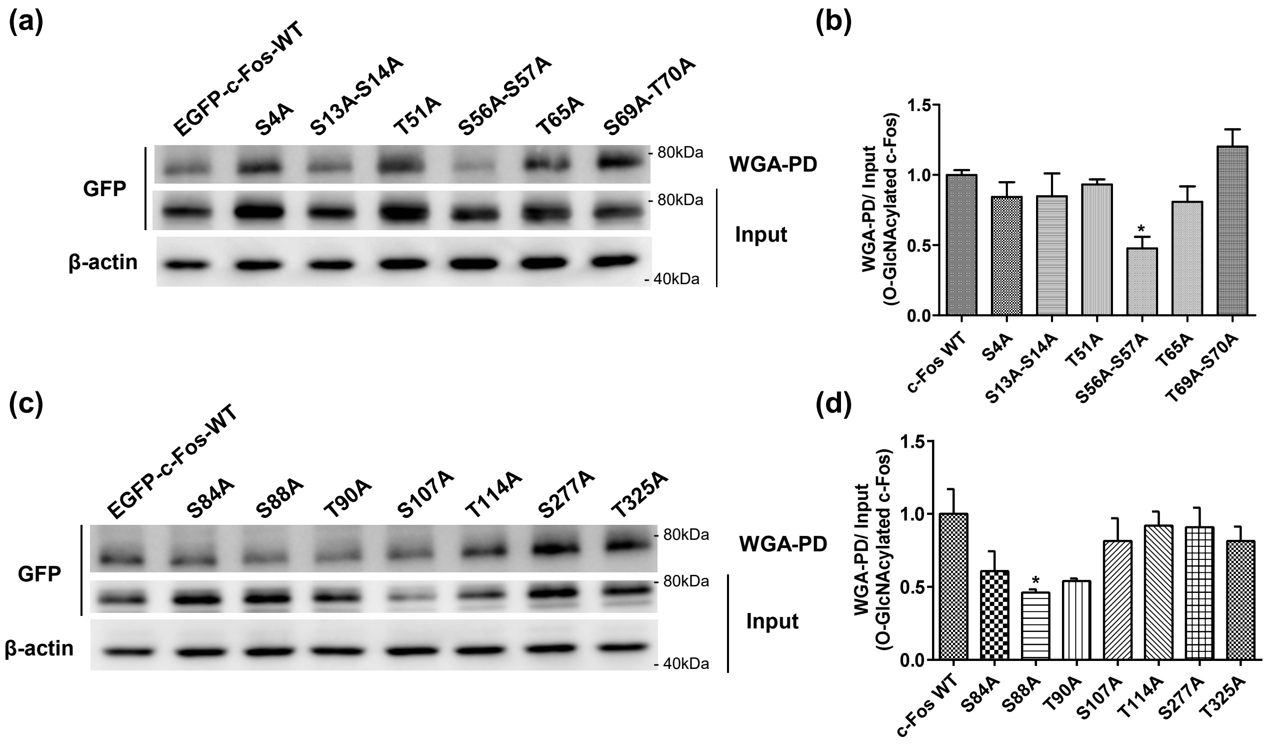

Figure S3. Identifying O-GlcNAc sites on c-Fos using a WGA-pull down assay

EGFP-c-Fos-WT and various EGFP-tagged potential O-GlcNAc silencing mutants, in which predicted O-GlcNAc sites (serine (S) or threonine (T)) were substituted with alanine (A), were WGA-pulled down and analyzed using Western blotting. (a, c) Representative immunoblot images. (b, d) Quantitative graph of c-Fos O-GlcNAcylation (n = 3). Data are presented as mean ± SEM. *$P < 0.05$ (one-way ANOVA, Bonferroni post-hoc test). WGA-PD: wheat-germ-agglutinin pull-down.

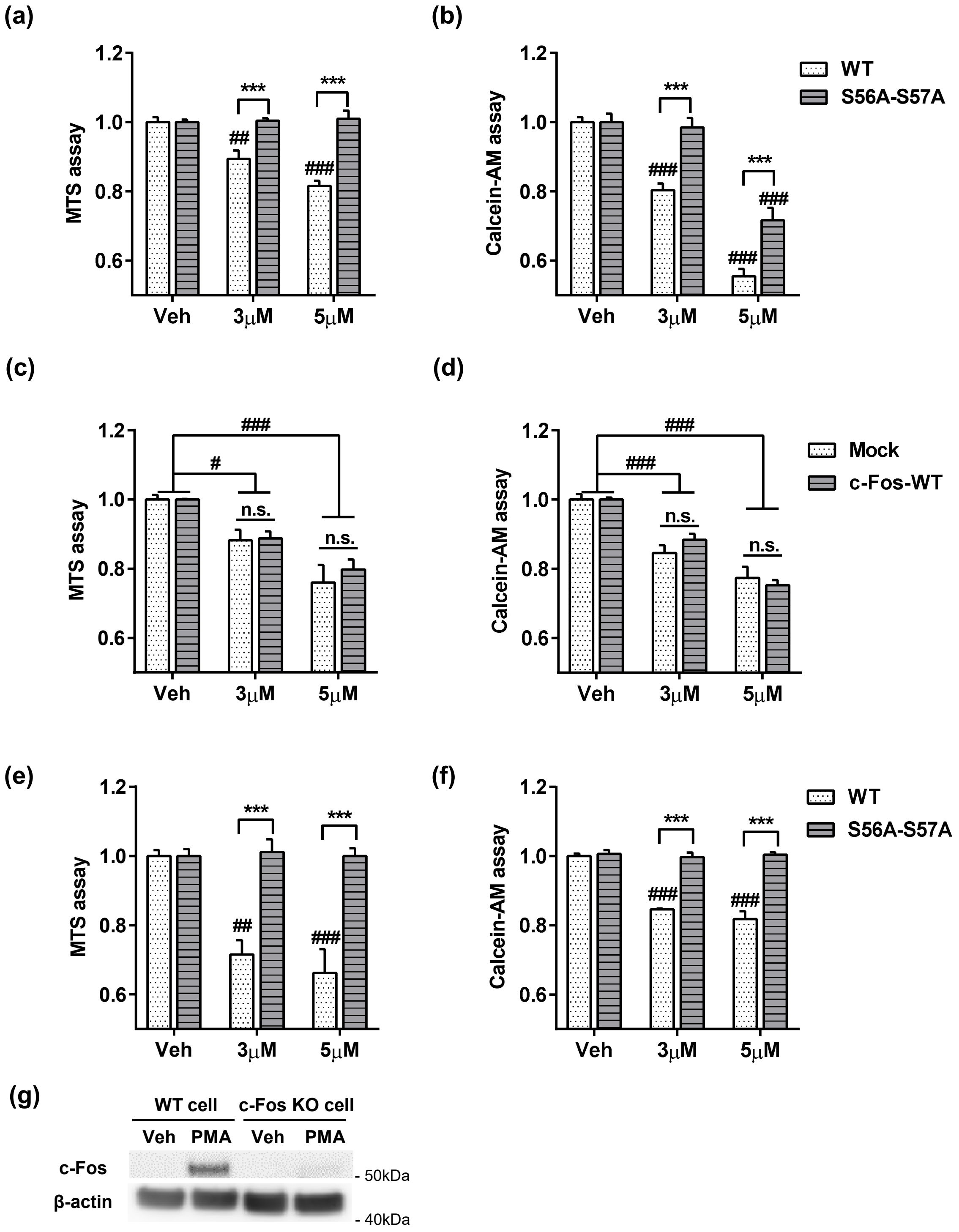

Figure S4. O-GlcNAcylation of c-Fos at S56 and S57 promotes cell death in the presence of Aβ

(a-b) Tag free-c-Fos-WT or tag free-c-Fos-S56A-S57A transfected HEK293T cells were treated with Aβ (at doses indicated) for 24 h. Quantitative graphs of MTS assay (a) and Calcein-AM assay (b) showing that tag free-c-Fos-S56A-S57A transfected groups are resistant to cell death compared to tag free-c-Fos-WT transfected groups in the presence of Aβ ((a): n = 8, (b): n = 8). (c-d) Mock vector and tag free-c-Fos-WT transfected SH-SY5Y cells were treated with Aβ (at doses indicated) for 24 h. Quantitative graphs of MTS assay (c) and Calcein-AM assay (d) showing that there were no significantly differences between Mock and c-Fos overexpression groups ((c): n = 6, (d): n = 6).

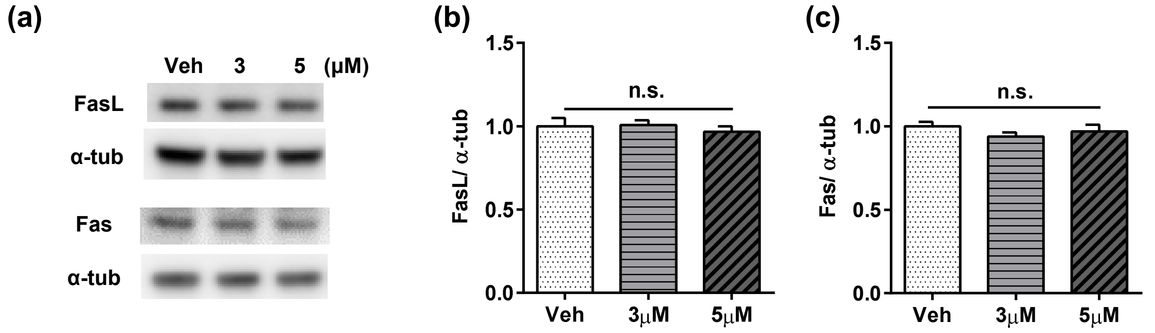

Figure S5. FasL and Fas, the target genes of AP-1, are not affected by Aβ in SH-SY5Y cells
SH-SY5Y cells were treated with Aβ (at doses indicated) for 24 h. (a) Representative immunoblot images (b-c) Quantitative graphs of FasL (b) and Fas (d) ((b): n = 4, (c): n = 4). Data are shown as mean ± SEM. (one-way ANOVA, Bonferroni post-hoc test). Veh: Vehicle, α-tub: α-tubulin, n.s.: non-significant.

Figure S4. O-GlcNAcylation of c-Fos at S56 and S57 promotes cell death in the presence of Aβ - Continued
(e-f) Tag free-c-Fos-WT or tag free-c-Fos-S56A-S57A transfected c-Fos knock-out SH-SY5Y cells were treated with Aβ (at doses indicated) for 24 h. Quantitative graphs of MTS assay (e) and Calcein-AM assay (f) showing that tag free-c-Fos-S56A-S57A transfected groups are resistant to cell death compared to tag free-c-Fos-WT transfected groups in the presence of Aβ, and that there was little interfering effect of endogenous c-Fos ((e): n = 7, (f): n = 6). (g) Confirmation of knocking-out c-Fos by Western blotting. Both regular wild-type cells and c-Fos knock-out cells were treated by PMA (200 nM, 3h) to induce c-Fos. Expectedly, c-Fos was not induced in c-Fos knock-out cells. β-actin was used as a loading control. Data are shown as mean ± SEM. # $P < 0.05$, ## $P < 0.01$, ### $P < 0.001$ among c-Fos-WT or c-Fos-S56A-S57A transfected groups (one-way ANOVA, Bonferroni post-hoc test), ***$P < 0.001$ between c-Fos-WT and c-Fos-S56A-S57A groups (two-way ANOVA, Bonferroni post-hoc test). Veh: Vehicle, WT: tag free-c-Fos-WT, S56A-S57A: tag free-c-Fos-S56A-S57A. n.s.: non-significant, WT cell: regular wild-type cell, c-Fos KO cell: c-Fos knock-out cell.

Supplemental Experimental Procedures

Construction of c-Fos knockout stable cell line

We adopted CRISPR/Cas9 system to knock-out c-Fos. There are three steps to construct c-Fos knockout stable cell line – 1) construction of CRISPR plasmid, which expresses Cas9-guided RNA (gRNA) targeting c-Fos, 2) transfection of CRISPR plasmid and Cas9 plasmid to SH-SY5Y cells, and 3) selection of c-Fos knockout cells using fluorescence-activated cell sorting (FACS) and antibiotics. For constructing CRISPR plasmid targeting c-Fos, we designed gRNA for c-Fos gene by using CRISPR design tool (http://crispr.mit.edu/), and cloned to gRNA empty vector (ID:41824, Addgene, USA) by using Gibson assembly (NEB, USA).(Mali et al., 2013) The sequences of gRNA targeting c-Fos are as follows – Forward: 5'-TTTCTTGGCTTTATATATCTTGTGGAAAGGACGAAACACC GGGGCTTCAACGCAGACTACGAGG-3', Reverse: 5'-GACTAGCCTTATTTTAACTTGCTATTTCTAGCTCTAAAAC CCTCGTAGTCTGCGTTGAAGCCCC-3'. For knocking-out c-Fos in SH-SY5Y cells, pEGFP-N1(Clontech, USA), hCas9 plasmid (ID:41815, Addgene, USA) and CRISPR plasmid which targeting c-Fos were co-transfected. After sorting GFP positive cells by FACS (BD, USA), cells were plated in 96 well plates with antibiotics (penicillin/streptomycin/neomycin; Sigma, USA) to select c-Fos knockout colonies. When colonies in each well were enough to subpassage, cells were transferred to 24 well plates, and tested c-Fos expression by western blotting. For inducing c-Fos expression, cells were treated with phorbol myristate acetate (PMA; Sigma, USA) before western blotting.

Supplemental References

Mali P, Yang L, Esvelt KM, Aach J, Guell M, DiCarlo JE, Norville JE, Church GM (2013). RNA-guided human genome engineering via Cas9. *Science*. **339**, 823-826.
